# Generation of Myeloid-Derived Suppressor Cells Mediated by MicroRNA-125a-5p in Melanoma

**DOI:** 10.3390/ijms25126693

**Published:** 2024-06-18

**Authors:** Samantha Lasser, Feyza Gul Ozbay Kurt, Lennart Fritz, Nina Gutzeit, Carolina De La Torre, Peter Altevogt, Jochen Utikal, Viktor Umansky

**Affiliations:** 1 Department of Dermatology, Venereology and Allergology, University Medical Center Mannheim, Heidelberg University, 68167 Mannheim, Germany; samantha.lasser@medma.uni-heidelberg.de (S.L.); feyza.kurt@medma.uni-heidelberg.de (F.G.O.K.); fritz_lennart@web.de (L.F.); ninagutzeit@gmail.com (N.G.); pe.altevogt@googlemail.com (P.A.); j.utikal@dkfz.de (J.U.); 2 Skin Cancer Unit, German Cancer Research Center (DKFZ), 69120 Heidelberg, Germany; 3 DFKZ-Hector Cancer Institute, University Medical Center Mannheim, 68167 Mannheim, Germany; 4 Mannheim Institute for Innate Immunoscience (MI3), Medical Faculty Mannheim, Heidelberg University, 68167 Mannheim, Germany; 5 NGS Core Facility, Medical Faculty Mannheim, Heidelberg University, 68167 Mannheim, Germany; carolina.delatorre@medma.uni-heidelberg.de

**Keywords:** microRNAs, extracellular vesicles, MDSCs, myeloid cells, melanoma

## Abstract

The ability of tumor-derived extracellular vesicles (EVs) to modulate the function of myeloid cells is widely recognized. Hence, a comprehensive understanding of the distinct components associated with EVs and the signals that they deliver to myeloid cells could provide potential approaches to impede the immunosuppression by myeloid-derived suppressor cells (MDSCs). We investigated melanoma EV-associated microRNAs (miRs) using the RET transgenic melanoma mouse model and simulated their transfer to normal myeloid cells by transfecting immature mouse myeloid cells and human monocytes. We observed elevated levels of miR-125a-5p, -125b-5p, and let-7e-5p in mouse melanoma-infiltrating MDSCs. In addition, miR-125a-5p levels in the tumor microenvironment correlated with mouse melanoma progression. The delivery of miR-125a-5p, alone or in combination with let-7e-5p and miR-99b-5p from the same genomic cluster, to normal myeloid cells resulted in their conversion to MDSC-like cells. Our findings indicate that miR-125a-5p could modulate myeloid cell activation in the melanoma microenvironment via a NF-κB-dependent mechanism.

## 1. Introduction

Melanoma development can often be attributed to exposure to mutagenic ultraviolet radiation and is frequently accompanied by a high tumor mutational burden [1]. This is believed to correlate with the presence of neoantigens, enabling immune recognition. Indeed, immune checkpoint inhibitor therapy to strengthen tumor-reactive immune cells has increased the median survival of melanoma patients significantly [2]. However, primary or acquired resistance mechanisms can limit their efficacy [3,4,5]. Immunosuppressive cells, such as myeloid-derived suppressor cells (MDSCs), may contribute to the failure of immunotherapies since MDSC levels in the peripheral blood of melanoma patients are higher in non-responders to immune checkpoint inhibitors than in responders [6,7,8].

MDSCs accumulate as a result of abnormal myelopoiesis, inadequate maturation of myeloid progenitors, and pathological activation of mature myeloid cells in a chronic inflammatory environment. Two major subtypes of MDSCs have been discovered: polymorphonuclear (PMN-) and monocytic (M-) MDSCs [9]. In humans, PMN-MDSCs are characterized as CD11b^+^CD14^−^CD15^+^ or CD11b^+^CD14^−^CD66b^+^ cells, while M-MDSCs are identified as CD11b^+^CD14^+^HLA-DR^−/lo^CD15^−^ cells [10]. In mice, MDSCs are analyzed based on the expression of Ly6G/Ly6C (Gr1), with PMN-MDSCs being CD11b^+^Ly6G^+^Ly6C^low^ and M-MDSCs being CD11b^+^Ly6G^−^Ly6C^high^ [10].

MDSCs are defined by their inhibitory function against other immune cells, especially T cells [10,11]. Multiple mechanisms underlie their immunosuppressive activity, including the expression of immune regulatory molecules such as programmed cell death ligand 1 (PD-L1) as well as the production of reactive oxygen species (ROS) or reactive nitrogen species via NADPH oxidase and inducible nitric oxide synthase (iNOS), respectively [12]. In addition, MDSCs can impair T cell functions through the activity of iNOS, arginase 1, and indoleamine 2,3-dioxygenase 1 (IDO1) [12].

Several tumor-derived growth factors, cytokines, and damage- or pathogen-associated molecular patterns can induce signaling pathways in myeloid cells, such as Toll-like receptor (TLR), mitogen-activated protein kinase (MAPK), nuclear factor κB (NF-κB), and janus kinase/signal transducer and activator of transcription (JAK/STAT) signaling, which regulate their activation state [13,14,15,16,17,18]. Soluble mediators, and also extracellular vesicle (EV)-associated molecules, can transmit such signals to myeloid cells in the tumor microenvironment (TME). For instance, soluble and EV-associated heat-shock protein HSP90α can induce MDSC-like cells through a TLR4/NF-κB axis [16,18]. Mouse macrophages have been shown to acquire tumor-promoting features as a response to EV-associated microRNA (miR)-21 and miR-29a interacting with endosomal TLR7 and TLR8 [19]. Additionally, sustained TLR stimulation in human monocyte-derived dendritic cells (DCs) could lead to immunosuppressive cell polarization through pathological MAPK, NF-κB, and STAT3 signaling [20]. Interestingly, this process was perpetuated by increased expression of the miR cluster 99b/let-7e/125a, which targeted negative regulators such as tribbles homolog 2 (TRIB2) and suppressor of cytokine signaling 1 (SOCS1).

MiRs are a group of non-coding RNAs that are highly conserved and have important functions in controlling gene expression at the post-transcriptional level [21,22,23,24]. Dysregulated expression of miRs has been linked to many human disorders, including cancer [25,26,27]. In melanoma, a set of eight miRs was identified to be enriched in EVs and also in monocytes exposed to melanoma EVs: miR-146a, miR-155, miR-125b, miR-100, let-7e, miR-125a, miR-146b, and miR-99b [28]. Monocytes stimulated with such EVs were converted into MDSCs, and antagonizing these miRs during exposure to melanoma EVs prevented their polarization toward MDSCs [28].

The effects of tumor EVs on myeloid cells have been explored previously [16,29,30,31]. We demonstrated that TLR activation by EV-associated HSP90α was one of the mechanisms, by which melanoma-derived EVs induced the conversion of normal myeloid cells to MDSCs [16]. However, the molecular processes, through which EV-associated miRs could contribute to MDSC generation in melanoma remained unknown. Therefore, we aimed here to elucidate the mechanisms induced by miRs, which have been shown to be associated with melanoma EVs [28]. We examined the abundance of such miRs in RET transgenic (tg) melanoma-bearing mice and found that miR-125a-5p is highly increased in tumor-infiltrating CD11b^+^Gr1^+^ cells. These cells are considered as MDSCs due to their previously shown immunosuppressive capacity [32,33,34]. Thus, we focused on analyzing how miR-125a-5p affects myeloid cell phenotype and function. To eliminate the complexity of EV cargo molecules, we mimicked miR transport by EVs using lipofection of synthetic miR-125a-5p. Such transfer of miR-125a-5p mimic to normal myeloid induced immunosuppressive functions of these cells via an NF-κB-dependent mechanism. Our study suggests that miR-125a-5p plays a critical role in the generation of MDSCs via melanoma EV-associated microRNA.

## 2. Results

### 2.1. MiR-125a-5p in the Mouse Melanoma Microenvironment

**Figure 1:**
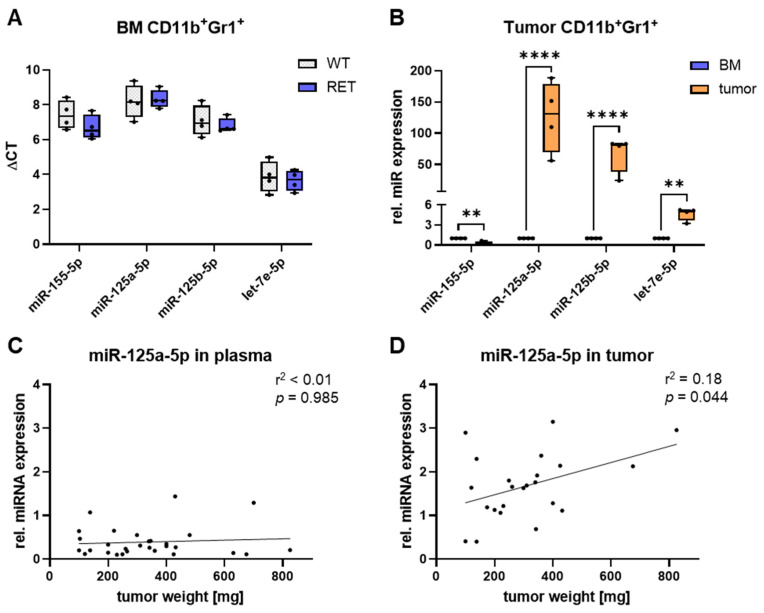
Amount of miR-125a-5p is enriched in tumor-infiltrating MDSCs and correlated with tumor progression in RET transgenic (tg) mice. (**A**) CD11b^+^Gr1^+^ cells were isolated from the bone marrow (BM) of healthy mice or tumor-bearing RET tg mice. The indicated miRs were measured by RT-qPCR (*n* = 4). (**B**) CD11b^+^Gr1^+^ cells were isolated from tumors of the same RET tg mice used in (**A**). The indicated miRs were measured by RT-qPCR, and their expression relative (rel.) to BM-derived CD11b^+^Gr1^+^ cells was calculated (*n* = 4). (**C**) MiR-125a-5p in plasma from tumor-bearing RET tg mice was measured by RT-qPCR, and its expression rel. to reference miRs was calculated (*n* = 29). (**D**) MiR-125a-5p in cutaneous tumors from RET tg mice was measured by RT-qPCR, and its expression rel. to reference miRs was calculated (*n* = 23). Box-and-whiskers plots: min to max; all data points are shown; the horizontal line is plotted at the median. Analysis of variance, linear regression analysis and Pearson correlation analysis were conducted using ΔCT values (** *p* < 0.01, **** *p* < 0.0001).

First, we studied the expression of miR-125a, -125b, -155, and let-7e in normal CD11b^+^Gr1^+^ immature myeloid cells (iMCs) and tumor-educated CD11b^+^Gr1^+^ iMCs, which can be designated as MDSCs. We found no differences between iMCs from the bone marrow (BM) of healthy wild-type (WT) mice and BM-MDSCs from tumor-bearing RET tg mice (Figure 1A). In contrast, tumor-infiltrating MDSCs exhibited higher levels of miR-125a, -125b, and let-7e than BM-MDSCs from the same RET tg mice (Figure 1B). Notably, miR-125a-5p was the most elevated miR in tumor-infiltrating MDSCs from RET tg mice, which was consistent with previous findings from our group in RET melanoma EV-treated iMCs [35]. Based on these observations, we hypothesized that miR-125a-5p may have the strongest impact on MDSC polarization among the melanoma EV-associated miRs. Hence, to further elucidate the role of this miR, we analyzed the association between miR-125a-5p levels and melanoma progression in the RET tg melanoma model. MiR-125a-5p abundance in the plasma of tumor-bearing RET tg mice was not associated with RET melanoma progression, that was determined by the weight of cutaneous tumors (Figure 1C). However, a positive correlation was identified between miR-125a-5p expression in the TME and RET melanoma progression (Figure 1D). Collectively, our findings pointed toward a particularly important role of miR-125a-5p in regulating the function of MDSCs within the TME.

### 2.2. Effects of Exogenous miR-125a-5p on Mouse Myeloid Cells

**Figure 2:**
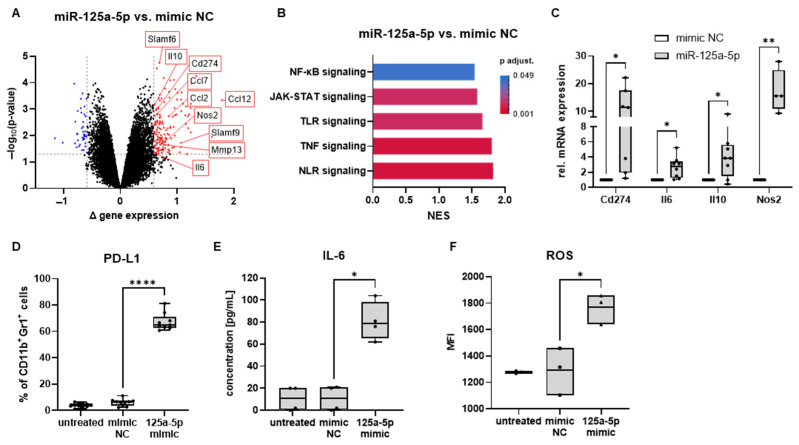
MiR-125a-5p induced characteristics of MDSCs in mouse iMCs. CD11b^+^Gr1^+^ iMCs from healthy mice were transfected with miR-125a-5p mimics or with miR mimic negative control molecules (mimic NC) and incubated for 20 h. Untreated iMCs were used as an additional control. (**A**) Differences in gene expression between miR-125a-5p-treated iMCs and mimic NC-treated iMCs were investigated by microarray analysis (*n* = 3). Black dots represent equivalent gene expression, blue dots indicate downregulated gene expression, and red dots indicate increased gene expression. MDSC-related genes were labeled. (**B**) Normalized enrichment scores (NES) for signaling pathways associated with changes in miR-125a-5p-treated iMCs identified by gene set enrichment analysis are shown. Adjusted *p*-values (*p* adjust.) are denoted by the color of the bars (*n* = 3). (**C**) Expression of the indicated genes was examined in miR-125a-5p-treated iMCs relative (rel.) to mimic NC-treated iMCs using RT-qPCR (*n* = 4–8). (**D**) PD-L1 expression on iMCs was measured by flow cytometry (*n* = 10). Results are shown as the percentage of PD-L1^+^ cells within total CD11b^+^Gr1^+^ iMCs. (**E**) Concentration of IL-6 in conditioned medium of iMCs was determined by ELISA and expressed in pg/mL (*n* = 4). (**F**) Production of ROS in iMCs was measured by flow cytometry using the CellROX deep red reagent (*n* = 3). Data are expressed as mean fluorescence intensity (MFI). Box-and-whiskers plots: min to max; all data points are shown; the horizontal line is plotted at the median. Paired two-tailed Student’s *t* test was performed (* *p* < 0.05, ** *p* < 0.01, **** *p* < 0.0001).

To investigate how miR-125a-5p could affect the polarization and function of myeloid cells, we examined changes in gene expression in iMCs transfected with miR-125a-5p mimic versus iMCs transfected with a miR mimic control. Microarray analysis revealed an increased expression of factors linked to MDSC function or recruitment in iMCs treated with miR-125a-5p mimic (Figure 2A). These factors included *Cd274* (encodes PD-L1), *Nos2* (encodes iNOS), *Mmp13*, *Il10*, *Il6*, *Ccl2*, *Ccl7*, and *Ccl12*. Additionally, elevated expression of *Slamf6* and *Slamf9*, which has been attributed to tumor-infiltrating myeloid cells [36,37], was observed in miR-125a-5p mimic-treated iMCs (Figure 2A).

A gene set enrichment analysis was conducted to gain more insights into the biological processes triggered by miR-125a-5p in iMCs. Annotated signaling pathways were examined to understand how miR-125a-5p may affect iMCs at a molecular level. Gene sets related to NF-κB, JAK-STAT, TLR, TNF, and NOD-like receptor signaling were enriched in miR-125a-5p mimic-treated iMCs (Figure 2B).

RT-qPCR confirmed a significant increase in the expression of *Cd274*, *Il6*, *Il10*, and *Nos2* in iMCs transfected with miR-125a-5p (Figure 2C). PD-L1 surface expression and IL-6 secretion by cells treated with miR-125a-5p mimics was measured by flow cytometry and ELISA, respectively. Gating strategies for iMCs are shown in Figure S1A. The levels of both markers were also markedly higher than in iMCs incubated with miR mimic negative control (Figure 2D,E). Moreover, ROS levels in iMCs transfected with miR-125a-5p mimics were significantly elevated as compared to those in control groups (Figure 2F and Figure S1B). In conclusion, miR-125a-5p mimic transfection induced the expression of factors associated with MDSC functions, such as PD-L1, IL-6, and ROS, suggesting that EV-associated miR-125a-5p could promote the pathological activation of myeloid cells in the melanoma microenvironment.

### 2.3. Mechanisms Underlying miR-125a-5p-Mediated Effects in Mouse Myeloid Cells

**Figure 3:**
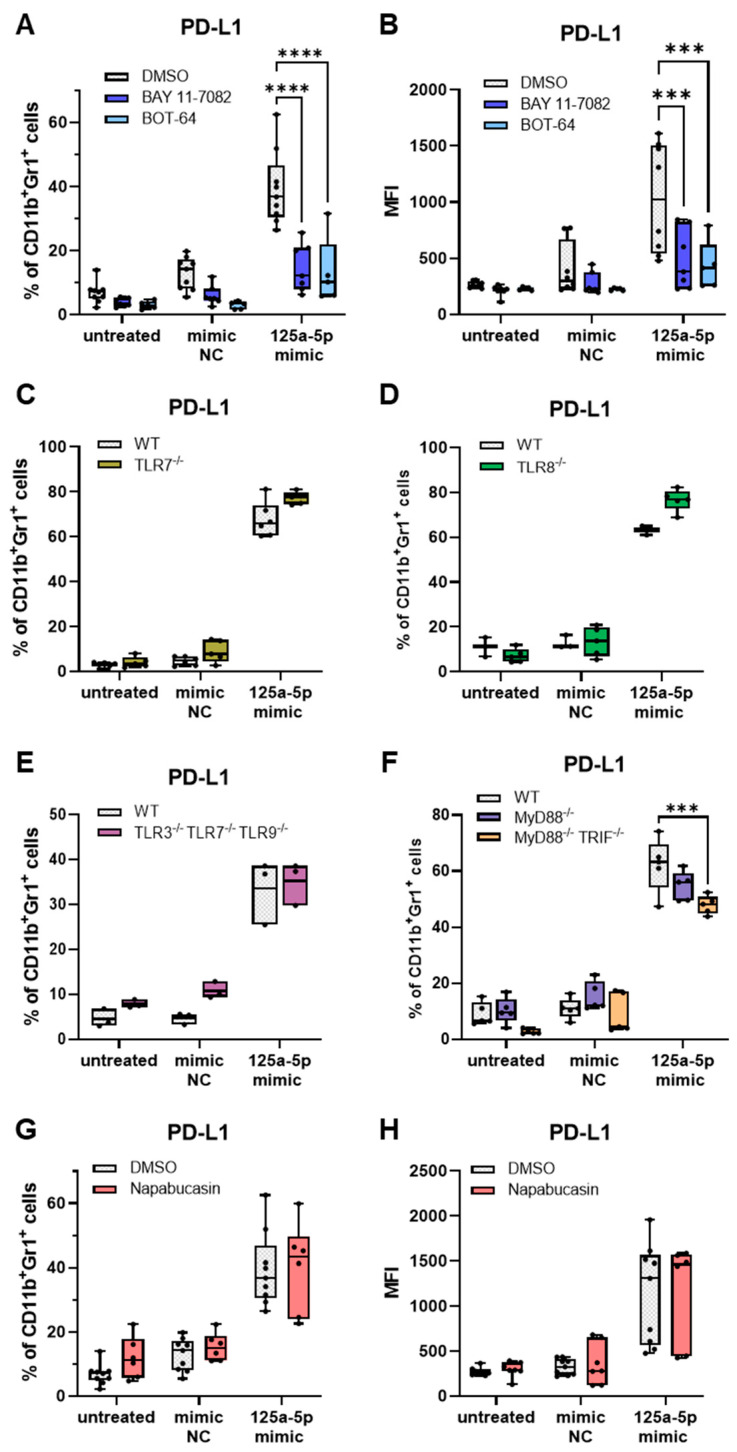
MiR-125a-5p-mediated upregulation of PD-L1 expression is dependent on NF-κB activity, but independent of endosomal TLRs and STAT3. CD11b^+^Gr1^+^ iMCs from healthy wild-type (WT) mice or different knock-out mice (as indicated) were transfected with miR-125a-5p mimics or miR mimic negative control molecules (mimic NC). Untreated iMCs were used as additional controls. PD-L1 expression in iMCs was measured after incubation for 20 h by flow cytometry. (**A**,**B**) Impact of NF-κB inhibitors (BAY-11-7082, BOT-64) on miR-125a-5p-induced PD-L1 expression (*n* = 5–6). Results are shown as (**A**) the percentage of PD-L1^+^ cells among total CD11b^+^Gr1^+^ iMCs or as (**B**) median fluorescence intensity (MFI). (**C**–**F**) Effect of impaired TLR signaling on miR-125a-5p-induced PD-L1 expression (*n* = 3–6). Data are presented as the percentage of PD-L1^+^ cells within total CD11b^+^Gr1^+^ iMCs. (**G**,**H**) Impact of STAT3 inhibition by napabucasin on miR-125a-5p-induced PD-L1 expression (*n* = 6). Results are shown as (**G**) the percentage of PD-L1^+^ cells among total CD11b^+^Gr1^+^ iMCs or as (**H**) MFI. Box-and-whiskers plots: min to max; all data points are shown; the horizontal line is plotted at the median. Analysis of variance and multiple comparisons were performed (*** *p* < 0.001, **** *p* < 0.0001).

The NF-κB signaling pathway is an important mechanism in the activation of MDSCs [15]. Thus, we explored its significance for the miR-125a-5p-mediated effects on mouse iMCs. The IKKβ inhibitors BAY-11-7082 and BOT-64 [38,39] were examined for their impact on MDSC-like polarization by miR-125a-5p, using PD-L1 expression as a functional marker. The blockage of NF-κB activation by either BAY-11-7082 or BOT-64, which prevent the phosphorylation of NF-κB inhibitor β, significantly diminished the upregulation of PD-L1 expression in miR-125a-5p-treated iMCs (Figure 3A,B).

Specific miRs have been identified as ligands for endosomal TLRs in myeloid cells, causing activation of NF-κB and contributing to the acquisition of tumor-promoting properties, including IL-6 production [19]. Thus, we investigated whether binding to endosomal TLRs is a potential mechanism for inducing the miR-125a-5p-mediated effects in iMCs. PD-L1 expression was assessed to evaluate the responsiveness of iMCs to miR-125a-5p in the presence or absence of endosomal TLRs. IMCs from TLR7^−/−^, TLR8^−/−^ or TLR3^−/−^TLR7^−/−^TLR9^−/−^ mice showed a response comparable to WT iMCs following miR-125a-5p transfection (Figure 3C–E). In addition, mice deficient in the adaptor molecules MyD88 and TRIF, which are critical for TLR downstream signaling [40], were used to exclude the possibility that TLR7 and TLR8 compensate for each other. PD-L1 expression in MyD88-deficient iMCs following miR-125a-5p transfection was similar to that in WT-iMCs (Figure 3F). MiR-125a-5p-treated iMCs deficient in both MyD88 and TRIF exhibited a slight decrease in PD-L1 expression (Figure 3F).

We next investigated whether STAT3 activation represented by the expression of pSTAT3 (Figure S2B) was involved in the miR-125a-5p-mediated effects since miR-125a-5p transfection triggered IL-6 production and the IL-6/STAT3 axis can be a strong driver of MDSC generation [41]. Napabucasin, a potent inhibitor of STAT3 activation, failed to affect the miR-125a-5p-induced PD-L1 expression in iMCs (Figure 3G,H).

### 2.4. MicroRNA Cluster 99b/let-7e/125a-Mediated Effects on Murine Myeloid Cells

Human monocyte-derived DCs were previously found to upregulate miR-125a, miR-99b and let-7e in response to sustained TLR stimulation, leading to a pathological activation of NF κB/STAT3 signaling by targeting negative regulators of MAPK and STAT3 [20]. MiR-125a, miR-99b and let-7e are expressed within one miR cluster, and multiple miRs in one cluster may cumulatively regulate the same process [42]. Notably, all three miRs were found to be present in EVs from melanoma patients [28]. Thus, we examined the combined effect of miR-125a-5p, miR-99b-5p and let-7e-5p (cluster) mimics on myeloid cells, hypothesizing that a direct transfer of the cluster miRs could regulate the NF-κB/STAT3 axis, bypassing the interaction between TLRs and TLR ligands.

**Figure 4:**
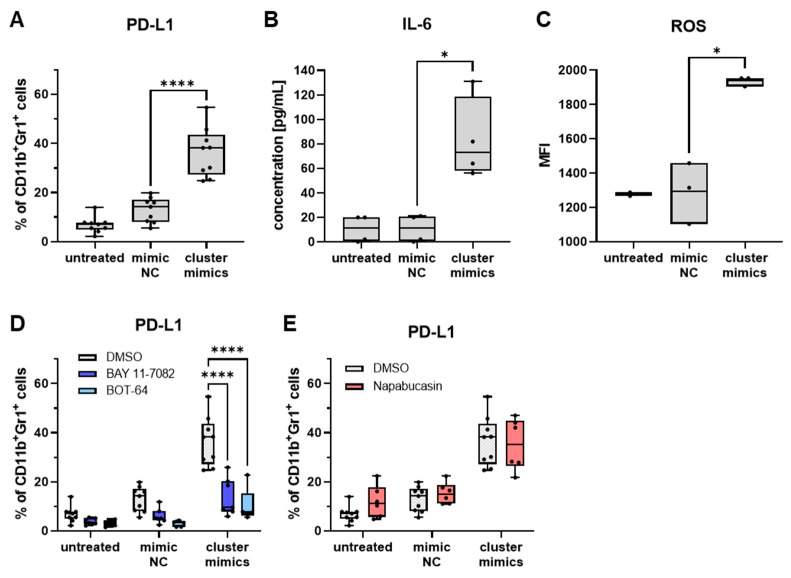
Investigation of miR cluster 99b/let-7e/125a-mediated effects on mouse iMCs. CD11b^+^Gr1^+^ iMCs from healthy mice were transfected with miR-125a-5p, miR-99b-5p and let-7e-5p (cluster mimics) or with miR mimic negative control (mimic NC) and incubated for 20 h. Untreated iMCs were used as an additional control. (**A**) PD-L1 expression on iMCs was measured by flow cytometry (*n* = 9). Results are shown as the percentage of PD-L1^+^ cells among total CD11b^+^Gr1^+^ iMCs. (**B**) Concentration of IL-6 in the conditioned medium of transfected iMCs was examined by ELISA and expressed as pg/mL (*n* = 4). (**C**) Production of ROS was determined by flow cytometry using the CellROX deep red reagent. Data are presented as median fluorescence intensity (MFI) (*n* = 3). (**D**) Impact of NF-κB inhibitors (BAY-11-7082, BOT-64) on miR-induced PD-L1 expression (*n* = 5–6). Results are shown as the percentage of PD-L1^+^ cells within total CD11b^+^Gr1^+^ iMCs. (**E**) Effect of the STAT3 inhibitor napabucasin on miR-induced PD-L1 expression (*n* = 6). Data are presented as the percentage of PD-L1^+^ cells among total CD11b^+^Gr1^+^ iMCs. Box-and-whiskers plots: min to max; all data points are shown; the horizontal line is plotted at the median. Analysis of variance and multiple comparisons were performed (* *p* < 0.05, **** *p* < 0.0001).

IMCs transfected with cluster mimics showed an elevation in PD-L1 expression and ROS production (measured by flow cytometry) as well as an increase in IL-6 secretion (measured by ELISA) as compared to iMCs transfected with a miR mimic control (Figure 4A–C). The effects mediated by the cluster mimics were equivalent to those exerted by miR-125a-5p mimic alone (Figure 2D–F), indicating that such modulation was mainly mediated by miR-125a-5p.

Blocking NF-κB activation with BAY 11-7082 or BOT-64 during transfection drastically diminished PD-L1 expression (Figure 4D). However, inhibiting STAT3 activation by napabucasin did not impede the upregulation of PD-L1 expression via cluster mimics. Overall, the findings suggested that miR-99b-5p and let-7e-5p had no additional effects on mouse iMCs as compared to the action of miR-125a-5p alone.

### 2.5. MicroRNA Cluster 99b/let-7e/125a-Mediated Effects on Human Monocytes

**Figure 5:**
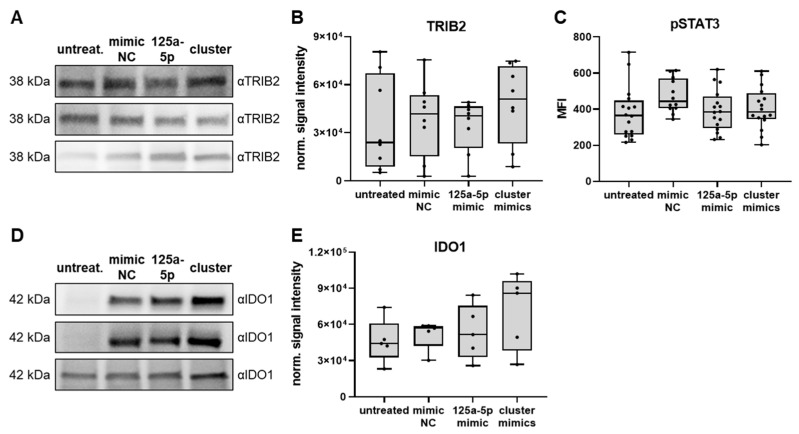
Investigation of mechanisms triggered by miR cluster 99b/let-7e/125a in human monocytes. CD14^+^ human monocytes were transfected with mimics of miR-125a-5p, miR-99b-5p, and let-7e-5p (cluster), miR-125a-5p mimics alone (125a-5p), miR mimic negative control molecules (mimic NC), or left untreated (untreat.). Analyses were performed after 48 h of incubation. (**A**) TRIB2 expression was measured by Western Blot analysis. Exemplary blots are shown for 3 biological replicates. (**B**) Signal intensity of TRIB2 staining normalized to the total amount of protein loaded is shown (*n* = 8). (**C**) STAT3 phosphorylation at tyrosine 705 (pSTAT3) was measured in monocytes by flow cytometry and shown as median fluorescence intensity (MFI). (**D**) IDO1 expression was measured by Western Blot analysis. Exemplary blots are shown for 3 biological replicates. (**E**) Signal intensity of IDO1 staining normalized to the total amount of protein loaded is shown (*n* = 5). Box-and-whiskers plots: min to max; all data points are shown; the horizontal line is plotted at the median. Analysis of variance and multiple comparisons were performed.

Considering the potential discrepancy between murine iMCs (comprising both polymorphonuclear and monocytic cells) and human monocytic cells, we studied whether direct delivery of miR-125a-5p, miR-99b-5p and let-7e-5p to human monocytes would alter their activation status via a MAPK/NF-κB/STAT3 axis similar to the effect of endogenous overexpression of this miR cluster published previously [20]. However, TRIB2, which regulates MAPK activity [43], was not significantly changed in healthy donor-derived monocytes transfected with miR-125a-5p mimic or cluster mimics as compared to mimic control-treated monocytes (Figure 5A,B). Furthermore, the transfection of miR-125a-5p mimic or cluster mimics had no impact on the levels of pSTAT3 (Figure 5C; the gating strategy for human monocytes is shown in Figure S2). This suggested that the transfected miRs did not cause a sustained STAT3 activation like endogenous overexpression of the miR cluster 99b/let-7e/125a. However, human monocytes transfected with cluster mimics tended to upregulate the expression of IDO1 (Figure 5D,E), which is an important effector molecule for MDSC immunosuppression. This could also be observed for monocytes treated with miR-125a-5p alone. However, the differences were not statistically significant.

**Figure 6:**
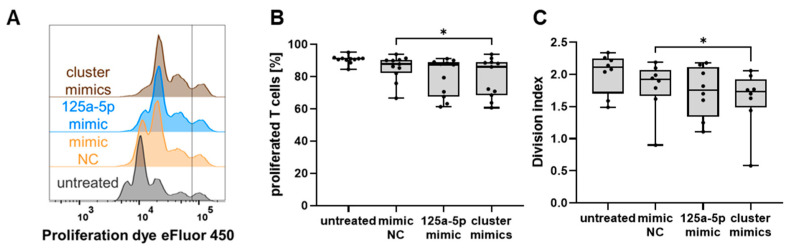
MiR-125a-5p, let-7e-5p, and miR-99b-5p induced immunosuppressive functions in human monocytes. Human T cells were labeled with a proliferation dye and co-cultured with transfected or non-transfected (untreated) monocytes, as indicated by the axis labels. T cell proliferation was determined by flow cytometry after 96 h of co-culture. (**A**) T cell proliferation was assessed based on the dilution of the proliferation dye as shown in the representative histograms. Each peak was defined as one generation. The vertical line marks the distinction between proliferated and non-proliferated cells. (**B**) The frequency of proliferated T cells is shown (*n* = 11). (**C**) The average number of divisions per T cell (division index) was calculated. Box-and-whiskers plots: min to max; all data points are shown; the horizontal line is plotted at the median. Analysis of variance and multiple comparisons were performed (* *p* < 0.05).

Next, T cell suppression assays were performed to test for functional changes in human monocytes transfected with miR-125a-5p or cluster mimics. Transfection with cluster mimics led to a modest reduction in T cell proliferation (Figure 6A,B). However, two donor-dependent groups became apparent: a cohort of monocytes on which the cluster miRs had no effect compared to control treatment, and another group of monocytes that demonstrated a clear suppressive activity against T cells upon exposure to cluster mimics. Additionally, when cluster mimics-transfected monocytes were co-cultured with T cells, the average number of divisions per T cell was significantly reduced (Figure 6C). Similar trends could be observed with miR-125a-5p mimic-stimulated monocytes (Figure 6B,C). Taken together, the data indicate that direct transfer of the cluster miRs may drive myeloid cells toward immunosuppressive MDSCs, which appears to be primarily mediated by miR-125a-5p. The responsiveness to the miRs, however, seems to be contingent on individual factors.

## 3. Discussion

It has been demonstrated that EVs originating from tumor cells can alter myeloid cell function by converting normal myeloid cells into MDSC-like cells [16,28,29,30,44]. Therefore, a thorough comprehension of the individual components associated with EVs and the signals they transmit to myeloid cells might offer strategies to interfere with MDSC immunosuppression and potentially enhance the effectiveness of immunotherapy. Huber et al. [28] have shown that melanoma EV-associated miRs could mediate MDSC generation. This study discovered that miR-146a, miR-155, miR-125b, miR-100, let-7e, miR-125a, miR-146b, and miR-99b were present in peripheral blood EVs isolated from melanoma patients. Furthermore, human monocytes treated with such melanoma EVs exhibited an increase in these miRs and acquired immunosuppressive functions [28]. In agreement with this, we found that mouse iMCs exposed to EVs obtained from RET melanoma cells displayed elevated levels of miR-146a, miR-155, miR-125b, let-7e, and miR-125a [35]. We could also show that tumor-infiltrating myeloid cells were enriched in miR-125b, let-7e, and miR-125a compared to myeloid cells in the bone marrow of the same RET transgenic mice (Figure 1B).

Among these miRs, miR-125a-5p was the most abundant miR in both EV-treated and tumor-infiltrating myeloid cells in the RET tg mouse melanoma model. Thus, we expected that miR-125a-5p could be the most relevant miR for the activation of tumor-promoting myeloid cell functions via EVs. This hypothesis was supported by a study reporting that alternatively activated macrophages (in vitro generated from mouse BM cells) displayed particularly high levels of miR-125a-5p [45]. In the RET transgenic mouse model of melanoma, miR-125a-5p levels in the TME correlated with tumor progression, as measured by tumor weight (Figure 1D). Interestingly, miR-125a was also found to be significantly enriched in the plasma of melanoma patients as compared to healthy donors [28].

Melanoma EVs were shown to promote the immunosuppressive polarization of myeloid cells by upregulating PD-L1 and iNOS expression, downregulating MHC II expression, as well as increasing IL-6, TNF-α, and TGF-β production [16,28,29,46]. To assess the contribution of miR-125a-5p as a cargo molecule to such effects and to study the underlying mechanisms, we produced vesicles containing solely the miR molecules using a lipid-based transfection reagent. An important criterion for selecting these vesicles to model EV transport was the pathway, by which they entered the cells. Endocytosis has been identified as the primary mechanism of uptake for both transfection vesicles and EVs, in particular by myeloid cells [47,48,49]. Thus, it was plausible that the miR delivered by transfection vesicles might elicit comparable signals in the cells as EV-associated miR. Indeed, a delivery of miR-125a-5p via transfection vesicles led to similar effects in mouse IMCs as a treatment with RET melanoma-derived EVs [16,28] such as the upregulation of PD-L1, IL-6, and ROS (Figure 2).

In previous research, we demonstrated that IL-6 could promote ROS production in mouse normal myeloid cells, thereby contributing to the immunosuppressive potential of these cells when converted to MDSCs [41]. Moreover, transcriptional analysis revealed an increase in IL-10, iNOS, and SLAM family members 6 and 9, which have been linked to tumor-associated myeloid cells [36,37]. Overall, the findings further support the hypothesis that miR-125a-5p might play a key role in the regulation of myeloid cells by melanoma EV-associated miR. Consistently, we observed reduced T cell proliferation in the presence of miR125a-5p-treated monocytes (Figure 6). In contrast, LPS/IFN-γ-stimulated mouse macrophages that were transfected with miR-125a have been reported to exert stimulatory effects on T cell proliferation [50].

Mechanistically, miR-125a-5p-mediated MDSC-like polarization appeared to strongly depend on NF-κB activity. Inhibition of NF-κB signaling substantially diminished PD-L1 expression in miR-125a-5p-treated iMCs (Figure 3A,B). Additionally, miR-125a-5p treatment led to increased expression of IL-6, IL-10, and iNOS (Figure 2C), which can be triggered by NF-κB [51,52,53]. Tumor necrosis factor alpha-induced protein 3 (TNFAIP3, also known as A20) has been identified as a direct target of miR-125a-5p [54,55,56,57], suggesting that miR-125a-5p could induce pathological activation of the NF-κB pathway by disturbing its negative regulation. Interestingly, NF-κB was reported to be a redox-sensitive signaling factor that can also be activated by ROS in myeloid cells [58,59,60]. MiR-125a-5p-transfected iMCs produced high amounts of ROS (Figure 2F), which might further sustain NF-κB activity.

Certain EV-associated miRs were shown to interact with endosomal TLRs [19]. Multiple studies demonstrated that TLR-mediated activation of NF-κB via MyD88 could promote MDSC immunosuppressive activity [13,14,61]. However, we did not observe an involvement of endosomal TLRs or MyD88 in the miR-125a-5p-mediated effects on iMCs (Figure 3C–F). On the other hand, miR-125a, let-7e, and miR-99b, which are organized in a genomic cluster, were reported to be elevated in human myeloid cells upon sustained TLR stimulation [20]. Upregulation of miR-125a, let-7e, and miR-99b expression caused prolonged MAPK/NF-κB and IL-6/STAT3 signaling in these cells by targeting TRIB2 and SOCS1, leading to the acquisition of immunosuppressive properties. In contrast, we failed to find any significant changes in TRIB2 and pSTAT3 levels when miR-125a-5p, let-7e-5p, and miR-99b-5p were delivered directly to human monocytes via transfection (Figure 5B,C), indicating that miR-125a-5p-mediated NF-κB activation, potentially via TNFAIP3 targeting, was the main mechanism in our system. However, future experiments are needed to verify this conclusion.

Overall, our findings shed light on the molecular processes by which melanoma EV-associated miRs could convert normal mouse and human myeloid cells into MDSCs. It involved the activation of the NF-κB pathway triggered by miR-125a-5p, resulting in transcriptional reprogramming of the cells and overexpression of MDSC effector molecules such as PD-L1. Our study provides further evidence that MDSCs are generated in melanoma not only by inflammatory mediators, but also by the reprogramming of normal myeloid cells via tumor-derived EVs, which may impair anti-tumor responses and, therefore, diminish the efficacy of melanoma immunotherapy.

## 4. Materials and Methods

### 4.1. Mice

*RET* tg mice (C57BL/6 background) that express the human *RET* oncogene in melanocytes under the control of the metallothionein-I promoter were initially provided by Dr. Izumi Nakashima (Chubu University, Aichi, Japan). All mice were bred and maintained under specific pathogen-free conditions at the animal facility of the German Cancer Research Center (DKFZ Heidelberg, Germany) and at the animal facility of the University Medical Center (Mannheim, Germany). Non-transgenic littermates served as healthy C57BL/6 control mice. The experiments were approved by the German local administration (approval number G-40/19) and conducted in compliance with ethical regulations. Femurs and tibias of TLR7-deficient (TLR7^−/−^) mice were kindly provided by Dr. Martina Seiffert (DKFZ Heidelberg, Germany). Femurs and tibias of TLR8-deficient (TLR8^−/−^) mice were kindly provided by Dr. Lena Alexopolou (Centre d’Immunologie de Marseille-Luminy, France). Femurs and tibias of TLR3/7/9-deficient (TLR3^−/−^TLR7^−/−^TLR9^−/−^) mice, MyD88-deficient (MyD88^−/−^) mice, and MyD88/TRIF-deficient (MyD88^−/−^TRIF^−/−^) mice were kindly provided by Dr. Carsten Kirschning (University Hospital Essen, Germany).

### 4.2. Blood and Tumor Sample Collection

Following CO_2_ asphyxia, 0.5 mL of blood was collected with an EDTA-containing syringe via cardiac puncture. The blood sample was centrifuged at 1900× *g* for 10 min to obtain 200–300 µL plasma. Cutaneous tumors were surgically removed, and the tumor weight was measured. All samples were snap frozen and stored at −80 °C until analysis.

### 4.3. Isolation of Mouse CD11b^+^Gr1^+^ Cells

CD11b^+^Gr1^+^ cells were isolated from femur and tibia bone marrow of C57BL/6 wild-type (WT) mice using immunomagnetic negative selection (EasySep™ Mouse CD11b+Gr1+ Isolation Kit, Stemcell Technologies, Vancouver, BC, Canada). Cutaneous tumors of *RET* tg mice were digested in DMEM supplemented with 5% (*v*/*v*) FBS (Thermo Fisher Scientific, Waltham, MA, USA), collagenase (1 mg/mL, Sigma-Aldrich, St. Louis, MO, USA), and DNase I (4 U/mL, Qiagen, Hilden, Germany) while being evenly shaken for 45 min at 37 °C. The cell suspension was filtered through a 100 µm cell strainer (Corning, Corning, NY, USA), and CD11b+Gr1+ cells were isolated by MACS^®^ cell separation (MDSC isolation kit mouse, Miltenyi Biotec, Bergisch Gladbach, Germany).

### 4.4. Isolation of Human Cells

Peripheral blood mononuclear cells (PBMCs) were separated from fresh healthy donor-derived buffy coats by density gradient centrifugation (Pancoll human, PAN-Biotech, Aidenbach, Germany). CD14^+^ monocytes and CD3^+^ T cells were isolated by MACS^®^ cell separation (CD14 microbeads and CD3 microbeads, Miltenyi Biotec).

### 4.5. MicroRNA Transfection

Myeloid cells (5.0 × 10^5^) were seeded in 100 µL RPMI medium 1640 GlutaMAX™ (Thermo Fisher Scientific) supplemented with 10% (*v*/*v*) FBS (Thermo Fisher Scientific). MiR mimics (30 pmol, Qiagen, Table S1) were diluted in Opti-MEM™ (100 µL, Thermo Fisher Scientific). HiPerFect transfection reagent (1 µL, Qiagen) was added to the diluted miR mimics. Transfection was performed according to the manufacturer’s protocol for suspension cells. When indicated, cells were incubated in the presence of 1 µM BAY-117082, 5 µM BOT-64, or 1 µM napabucasin after addition of the miR complexes. Respective amounts of DMSO were used as solvent control.

### 4.6. RNA Isolation

iMCs (3.0 × 10^6^) were stimulated with miR mimics for 20 h and used for total RNA isolation with the RNeasy Mini Kit (Qiagen). On-column DNAse digestion was performed using the RNAse-free DNase set (Qiagen). For miR isolation from tumor tissue or tumor-infiltrating cells, the miRNeasy Mini Kit (Qiagen) was used. For miR isolation from plasma, the miRNeasy Serum/Plasma Kit (Qiagen) was used.

### 4.7. RT-qPCR

RNA (500 ng) was used for reverse transcription of mRNA using the SensiFAST™ cDNA synthesis kit (Bioline, Little Clacton, UK) according to the manufacturer’s instructions. Quantitative PCR was performed using the SensiFAST™ SYBR^®^ Lo-ROX Kit (Bioline) and primers purchased from Metabion (Table S2). Rn18s was used for normalization. Reverse transcription reactions for miR quantification were set up with the miRCURY LNA RT Kit (Qiagen) according to the manufacturer’s instructions for tissue or plasma samples. The RNA spike-in kit (Qiagen) was employed. Quantitative PCR was performed using the miRCURY LNA SYBR^®^ Green PCR Kit (Qiagen) and the following miRCURY LNA miRNA PCR assays (Qiagen) were used for normalization: hsa-miR-125a-5p, hsa-miR-125b-5p, hsa-let-7e-5p, mmu-miR-155-5p, hsa-miR-103a-3p, cel-miR-39-3p, and UniSp6. MiR-103-3p, miR-39-3p, and UniSp6.

### 4.8. Microarray Analysis

Transcriptome profiling was conducted by the microarray core facility of the DKFZ Heidelberg. Further analysis was conducted by the NGS Core Facility and Medical Faculty Mannheim (University of Heidelberg). The GeneChip™ Mouse Gene 2.0 ST Array (Thermo Fisher Scientific) was used. A Custom CDF V.24 with ENTREZ-based gene definitions was applied to annotate the arrays [62]. The raw fluorescence intensity values were normalized by quantile normalization and robust multiarray analysis background correction. One-way analysis of variance was performed to identify differentially expressed genes using the commercial software package SAS JMP15 Genomics V.10. Ulterior gene set enrichment analysis was conducted [63] to determine if defined lists of genes exhibit a statistical bias in their distribution within a ranked gene list. Pathways belonging to various cell functions such as cell cycle or apoptosis were obtained from public external databases (KEGG, http://www.genome.jp/kegg accessed on 27 May 2021).

### 4.9. Flow Cytometry

For extracellular staining, 2 × 10^5^ cells/test were incubated with Fc Block™ (BD Biosciences, Franklin Lakes, NJ, USA) for 10 min at 4 °C. Subsequently, the following monoclonal antibodies were added for 20 min at 4 °C: CD11b-APC-Cy7 (clone M1/70, BD Biosciences), Gr1-PE-Cy7 (clone RB6-8C5, BD Biosciences), PD-L1-BV421 (clone MIH5, BD Biosciences), or isotype control BV421 (BD Biosciences). 7-AAD staining solution (Miltenyi Biotec) and CellROX^®^ Deep Red reagent (Thermo Fisher Scientific) were used according to the manufacturers’ instructions. For intracellular staining, 5 × 10^5^ cells/test were blocked with FcR blocking reagent (Miltenyi Biotec) and stained with fixable viability dye 700 (BD Biosciences), CD14-FITC (clone MΦP9, BD Biosciences), and Stat3 (pY705)-PE (clone 4/P-STAT3, BD Biosciences) using the Phosflow™ Fix buffer I (BD Biosciences) and the BD Phosflow™ Perm buffer III (BD Biosciences). Data were acquired with the BD FACSLyric flow cytometer and the FACSuite software v.1.5 (BD Biosciences). Data analysis was performed with the FlowJo v.10 software (BD Biosciences).

### 4.10. T Cell Suppression Assay

The assay was carried out in accordance with the standardized Mye-EUNITER protocol [64]. Briefly, human CD3^+^ T cells were labeled with cell proliferation dye eFluor 450 (10 µM, eBioscience, San Diego, CA, USA) and seeded in 96-well round-bottom plates (Sarstedt) coated with anti-CD3 (1 µg/mL, clone OKT-3, eBioscience) and anti-CD28 antibodies (2 µg/mL, clone CD28.2, Beckman Coulter, Brea, CA, USA). Allogeneic CD14^+^ monocytes stimulated with miR mimics for 72 h in the presence of GM-CSF (40 ng/mL, InvivoGen, San Diego, CA, USA) were added. T cell proliferation was measured as a dilution of the cell proliferation dye using the BD FACSLyric flow cytometer and the FACSuite software v.1.5 (BD Biosciences). Proliferation analysis was performed using the FlowJo v.10 software (BD Biosciences).

### 4.11. ELISA

The IL-6 concentration was measured in the supernatants of iMCs treated with miR mimics for 24 h using the ELISA MAX Deluxe Set Mouse IL-6 (BioLegend, San Diego, CA, USA) according to the manufacturer’s instructions.

### 4.12. Western Blot

Monocytes (2.5 × 10^6^) were harvested 48 h after transfection and lysed in RIPA buffer (Thermo Fisher Scientific), 1×x protease inhibitor (Promega, Madison, WI, USA), 1× sodium fluoride (Jena Bioscience, Jena, Germany), sodium orthovanadate (1 µM, Jena Bioscience) for 30 min on ice. Lysates were centrifuged at 16,000× *g* and 4 °C for 15 min. Supernatants were mixed with 4× NuPAGE™ LDS sample buffer (Thermo Fisher Scientific), heated to 95 °C for 5 min, loaded onto 4–15% Mini-PROTEAN protein gels (Bio-Rad, Hercules, CA, USA) and blotted onto a PVDF membrane (Thermo Fisher Scientific). The PVDF membrane was blocked in TBS buffer, 0.1% Tween 20 (Bio-Rad), 3% (*w*/*v*) BSA (Carl Roth, Karlsruhe, Germany) and incubated with primary antibodies (1:1000 in blocking buffer) for 1 h at room temperature (anti-human IDO-1, D5J4E, Cell Signaling; anti-human TRIB2, Abcam, kindly provided by Dr. Dagmar Hildebrand, University Hospital Heidelberg, Germany). Following a washing step, the membrane was incubated with secondary antibodies (1:10,000 in blocking buffer) for 1 h at room temperature (peroxidase-conjugated goat anti-rabbit IgG, Jackson ImmunoResearch, West Grove, PA, USA). Following a washing step, chemiluminescence was measured using Pierce ECL Western Blotting Substrate (Thermo Fisher Scientific), imaging system Fusion SL4 (Viber Lourmat, Collégien, France), and FusionCapt Advance SL4 (Viber Lourmat). ImageJ v.1.53s was used to determine the signal intensity, which was normalized to the total amount of protein loaded.

### 4.13. Statistical Analysis

Statistical analysis of data was performed using GraphPad Prism software v.9 on at least three biological replicates. Two groups were compared with paired two-tailed Student’s *t* test. Analysis of variance and multiple comparisons were applied for more than two groups. Correlation analysis was carried out using Pearson correlation with two tailed *p* value. A *p* value of <0.05 was considered statistically significant.

## Data Availability

The data presented in this article will be made available by the corresponding author on request.

## Supplementary Materials

The following supporting information can be downloaded at: https://www.mdpi.com/article/10.3390/ijms25126693/s1.
