# Supplementary material for: Generation of Myeloid-Derived Suppressor Cells Mediated by MicroRNA-125a-5p in Melanoma
Source: Int J Mol Sci. 2024 Jun 18;25(12):6693. doi: 10.3390/ijms25126693 (PMC11203613; doi:10.3390/ijms25126693)
Supplement: Supplementary file 1 [file ijms-25-06693-s001.zip › ijms-2963003-supplementary.pdf]

Table S1. MicroRNA mimics used for transfection experiments.

miRCURY LNA microRNA mimic	Sequence (5' to 3')
hsa-miR-125a-5p	UCCCUGAGACCCUUUAACCUGUGA
hsa-let7e-5p	UGAGGUAGGAGGUUGUAUAGUU
hsa-miR-99b-5p	CACCCGUAGAACCGACCUUGCG
Negative control	GAUGCUACGGUCAAUGUCUAAG

Table S2. Primers used for RT-qPCR.

		Sequence (5' to 3')
**18S ribosomal RNA**	forward	CGCGGTTCTATTTTGTTGGT
	reverse	AGTCGGCATCGTTTATGGTC
**Il6**	forward	TTCCATCCAGTTGCCTTC TTG
	reverse	GAAGGCCGTGGTTGTCACC
**Il10**	forward	ATAACTGCACCCACTTCCCA
	reverse	GGGCATCACTTCTACCAGGT
**CD274**	forward	TGGACAAACAGTGACCACCAA
	reverse	CCCCTCTGTCCGGGAAGT
**Nos2**	forward	TTGGGTCTTGTTAGCCTAGTC
	reverse	TGTGCAGTCCCAGTGAGGAAC

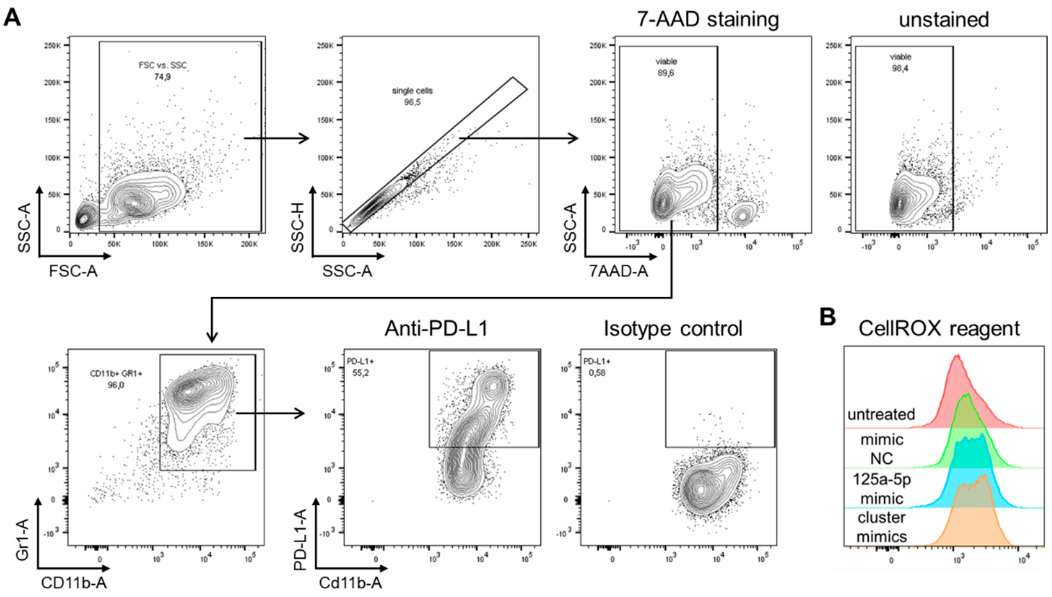

Figure S1. Gating strategy for marker expression on immature myeloid cells (iMCs). (**A**) CD11b^{+}Gr1^{+} iMCs were gated after exclusion of cell debris, doublets, and dead cells. PD-L1^{+} iMCs were gated according to the respective isotype control. (**B**) Representative histograms for the expression of reactive oxygen species (ROS) in live iMCs using the CellROX reagent.

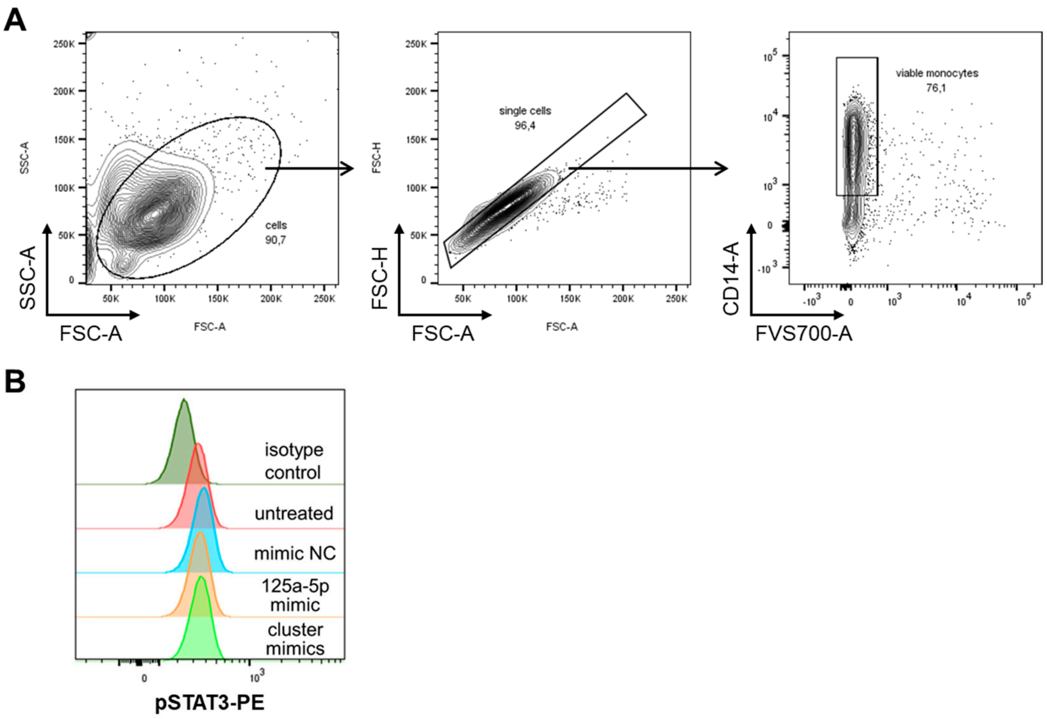

Figure S2. Gating strategy for human monocytes. (**A**) After excluding cell debris and doublets, monocytes were gated according to their CD14 expression and negative staining for fixable viability 700 (FVS700). (**B**) Representative histograms for the expression of phosphorylated STAT3 (Y705) in live monocytes.
